# Antitumor Efficacy of the Dual PI3K/mTOR Inhibitor PF-04691502 in a Human Xenograft Tumor Model Derived from Colorectal Cancer Stem Cells Harboring a *PIK3CA* Mutation

**DOI:** 10.1371/journal.pone.0067258

**Published:** 2013-06-27

**Authors:** Douglas D. Fang, Cathy C. Zhang, Yin Gu, Jitesh P. Jani, Joan Cao, Konstantinos Tsaparikos, Jing Yuan, Melissa Thiel, Amy Jackson-Fisher, Qing Zong, Patrick B. Lappin, Tomoko Hayashi, Richard B. Schwab, Anthony Wong, Annette John-Baptiste, Shubha Bagrodia, Geritt Los, Steve Bender, James Christensen, Todd VanArsdale

**Affiliations:** 1 Oncology Research Unit, Pfizer Global Research & Development, San Diego, California, United States of America; 2 Drug Safety Research and Development, Pfizer Global Research & Development, San Diego, California, United States of America; 3 Moores UCSD Cancer Center, University of California San Diego, San Diego, California, United States of America; Seoul National University, Republic of Korea

## Abstract

*PIK3CA* (phosphoinositide-3-kinase, catalytic, alpha polypeptide) mutations can help predict the antitumor activity of phosphatidylinositol-3-kinase (PI3K)/mammalian target of rapamycin (mTOR) pathway inhibitors in both preclinical and clinical settings. In light of the recent discovery of tumor-initiating cancer stem cells (CSCs) in various tumor types, we developed an *in vitro* CSC model from xenograft tumors established in mice from a colorectal cancer patient tumor in which the CD133+/EpCAM+ population represented tumor-initiating cells. CD133+/EpCAM+ CSCs were enriched under stem cell culture conditions and formed 3-dimensional tumor spheroids. Tumor spheroid cells exhibited CSC properties, including the capability for differentiation and self-renewal, higher tumorigenic potential and chemo-resistance. Genetic analysis using an OncoCarta™ panel revealed a *PIK3CA (H1047R)* mutation in these cells. Using a dual PI3K/mTOR inhibitor, PF-04691502, we then showed that blockage of the PI3K/mTOR pathway inhibited the *in vitro* proliferation of CSCs and *in vivo* xenograft tumor growth with manageable toxicity. Tumor growth inhibition in mice was accompanied by a significant reduction of phosphorylated Akt (pAKT) (S473), a well-established surrogate biomarker of PI3K/mTOR signaling pathway inhibition. Collectively, our data suggest that PF-04691502 exhibits potent anticancer activity in colorectal cancer by targeting both *PIK3CA (H1047R)* mutant CSCs and their derivatives. These results may assist in the clinical development of PF-04691502 for the treatment of a subpopulation of colorectal cancer patients with poor outcomes.

## Introduction

Colorectal cancer is the third most commonly diagnosed cancer for both men and women in the United States. Approximately 103,170 new cases of colorectal cancer are diagnosed yearly, with about 51,690 annual deaths [1]. In colorectal cancer, the PI3K/mTOR pathway is frequently dysregulated because of mutations in the p110α subunit of *PI3K*. The PI3K/mTOR signaling pathway is a key regulator of various cell processes, including proliferation, differentiation, apoptosis, motility, metabolism, and autophagy. Thus, oncogenic pathway activation has become an attractive therapeutic target for drug development [2]. Preclinical and clinical studies indicate that the *PIK3CA* mutation in the absence of a *KRAS* mutation is a predictive marker for the response to PI3K and mTOR inhibitors [3]–[6].

In addition to the recently launched mTOR inhibitor everolimus, which is used for the treatment of a broad range of cancer types, a number of small molecule inhibitors of the PI3K/mTOR signaling pathway are currently in clinical development [7]–[10]. These agents include PI3K-selective inhibitors, AKT inhibitors, mTOR catalytic site inhibitors, and dual PI3K/mTOR inhibitors. PF-04691502, 2-amino-8-[trans-4-(2-hydroxyethoxy)cyclohexyl]-6-(6-methoxypyridin-3-yl)-4-methylpyrido[2,3-d]pyrimidin-7(8H)-one, is a potent dual inhibitor of all PI3K isoforms and mTOR (TORC1 and TORC2). The pharmacological attributes of this orally efficacious agent were recently reported [11]. PF-04691502 potently inhibited recombinant class I PI3K and mTOR in biochemical assays and suppressed the transformation of avian fibroblasts mediated by wild type PI3Kγ, δ or mutant PI3Kα. PF-04691502 also inhibited mTORC1 activity in cells. In *PIK3CA*-mutant cancer cell lines, PF-04691502 suppressed the phosphorylation of AKT (S473) and inhibited cell proliferation [11]. PF-04691502 demonstrated antitumor activities in ovarian and glioblastoma tumor xenograft models derived from cancer cell lines carrying either a *PTEN* deletion or *PIK3CA* mutation [11] and in a genetically engineered mouse model of ovarian cancer driven by a *Kras* mutation and *Pten* deletion [12]. The antitumor activities of PI3K/mTOR inhibitors, including PF-04691502, in colorectal cancer remain to be explored.

Recent studies have demonstrated that the hierarchically organized colorectal tumors compose a small subset of CD133+/EpCAM+ expressing CSCs [13], [14]. CSCs are essential for tumor development and progression, as well as drug resistance and tumor metastasis [15], [16]. The escape of CSCs from current chemo- and radiotherapies could explain resistance and tumor relapse [15], [16]. The importance of the PI3K/mTOR signaling network in CSC biology has been noted recently [17]. The correlation of AKT activation and increased tumorigenicity, stemness, and invasiveness was identified in a glioblastoma model [18]. Activated PI3K signaling was found to play a crucial role in the tumorigenesis of prostate basal/stem cells [19], [20]. In combination with other agents, the blockage of PI3K/mTOR pathway in pancreatic cancer was able to eliminate pancreatic CSCs and leukemia stem cells, demonstrating the anti-CSC efficacy of inhibiting the PI3K/mTOR pathway [21], [22]. Considering the important functions of CSCs, it is interesting to explore whether therapeutic agents targeting the PI3K/mTOR signaling pathway are effective in colorectal cancer driven by CSCs harboring a somatic *PIK3CA* mutation.

We report here the oral efficacy of a dual PI3K/mTOR inhibitor, PF-04691502, in a human colon cancer xenograft model driven by highly tumorigenic CD133+/EpCAM+ CSCs. In the patient tumor, CD133+/EpCAM+ cells represented the tumor-initiating cells upon xenotransplantation. CD133+/EpCAM+ cells were then propagated and enriched as tumor spheroid cells under stem cell conditions. Tumor spheroid cells possessed the additional characteristics of CSCs, including self-renewal, chemo-resistance, higher tumorigenic potential *in vivo*, and the capacity to recapitulate the histopathological features of the original patient tumor *in vivo*. Genetic analysis of 19 common oncogenes revealed that the CSC population being tested harbored a *PIK3CA (H1047R)* mutation. Furthermore, a dual PI3K/mTOR inhibitor, PF-04691502, markedly inhibited the proliferation of CSCs *in vitro,* as well as the growth of xenograft tumors derived by the CSC population in mice. Western blot analysis showed that pAKT (S473) was downregulated in xenograft tumors that were treated with PF-04691502. Overall, our results suggest that the PI3K/mTOR pathway inhibitor PF-04691502 has potent anti-proliferative activity in *PIK3CA* mutant CSCs, warranting further evaluation of the inhibitors in colorectal cancer patients carrying PIK3CA mutations.

## Materials and Methods

### Animals, Patient Sample, and the Establishment of Patient-derived Xenograft (PDX) Tumor Model

Written informed consent was obtained from the study participant, a 39-year-old man with colorectal cancer, prior to surgery and the collection of the tissue sample. The procedures were approved by the University of California at San Diego Human Research Protections Program Institutional Review Board (IRB). All experimental animal procedures complied with the Guide for the Care and Use of Laboratory Animals (Institute for Laboratory Animal Research, 1996) and were approved by the Pfizer Global Research and Development Institutional Animal Care and Use Committee. Briefly, resection revealed a stage T3N2 poorly differentiated colorectal mucinous carcinoma with signet-ring cell features. Surgically resected tumor tissue was cut into 2- to 4-mm^3^ fragments and subcutaneously implanted into the flank of NOD/SCID mice (The Jackson Laboratory, Bar Harbor, Maine). Palpable P_0_ xenograft tumors (the first generation in mice) formed in mice at six weeks post-implantation. When the size of tumors reached approximately 500 mm^3^, P_0_ tumors were then expanded in ten NOD/SCID mice by subcutaneous re-implantation of the tumor fragments to generate P_1_ xenograft tumors. The histopathology of the xenograft tumors was reviewed in comparison with the patient tumor by a board certificated pathologist (PBL).

### Tissue Processing and Tumor Spheroid Cultures of CSCs

Surgically removed xenograft tumor tissues from mice were enzymatically dissociated using Accumax solution (Sigma-Aldrich, St Louis, MO, USA) into single cells and cultured in serum-free stem cell medium at 37°C in a 5% CO_2_, humidified atmosphere. Stem cell medium was generated by conditioning serum-free culture medium, which was routinely used for human embryonic stem cells (hESC), in mouse embryonic fibroblast cultures for 24 hrs and then mixing the conditioned medium with fresh hESC medium (at a 1∶1 ratio) supplemented with 4 ng/ml bFGF, 10 ng/ml EGF, 10 mg/ml bovine insulin (Sigma-Aldrich, St Louis, MO, USA), 5.5 mg/ml human transferrin (Sigma-Aldrich), and 5 ng/ml sodium selenite (Sigma-Aldrich; 23). Primary tumor cells were cultured in Ultra-low attachment surface flasks (Corning, Lowell, MA) for the first 3 months to form nonadherent tumor spheroids. After spheroid cell cultures became stable, CSC cultures were transferred to Nunc™ non-treated polystyrene cell culture flasks (Thermo Fisher Scientific, Rochester, NY).

### Flow Cytometry and Fluorescence-activated Cell Sorting (FACS)

Standard cell surface antigen labeling and flow cytometry evaluations were performed. Dissociated tumor cells were analyzed using phycoerythrin-conjugated CD133 (Miltenyi Biotech) and APC-conjugated epithelium-specific antigen EpCAM (CD326, epithelial cell surface antigen, BD Biosciences) antibodies. Cell sorting was performed on a FACSAria I cell sorter (BD). Unstained cells and cells stained with isotype-matched control antibodies were used as negative controls. In primary cells isolated from xenograft tumors, dead cells and murine cells were excluded using propidium iodide (PI) and an anti-mouse specific monoclonal antibody conjugated with fluorescein isothiocyanate (H-2k[d]-FITC; BD), respectively.

### Cell Viability Assay after Treatment with Compound

Dissociated viable, single spheroid CSCs and differentiated cells were plated at 10,000 and 5,000 cell/well, respectively, in clear bottom 96-well plates (Corning) and treated with the indicated compounds for 5 days. Cell viability was determined by using a CellTiter Glo® luminescent cell viability assay kit (Promega).

### Cell Proliferation and Differentiation Induction

Cell proliferation and cytokeratin expression were measured simultaneously using a fluorescein isothiocyanate 5-bromodeoxyuridine (BrdU) flow kit (BD Biosciences) and anti-cytokeratin antibodies (phycoerythrin-conjugated cytokeratin 7/8 CAM 5.2, BD Biosciences). CSC differentiation was induced by culturing spheroid CSCs in DMEM medium supplemented with 10% FBS (Invitrogen, Carlsbad, CA) in regular cell culture flasks.

### Mutational Analysis of Oncogenic Mutations

Mutational sequencing was provided by Sequenom (www.sequenom.com) using an OncoCarta™ panel v1.0, which contains 238 hotspot mutations in 19 oncogenes within 24 multiplexes. The results were analyzed using the Oncomutations Report function in Typer 4.0 (Sequenom Inc, San Diego, USA) using the function “OncoMutation Reports”. This algorithm reprocesses the raw spectral data and automatically compensates peak areas based on previously established salt and matrix adduct formations that can obscure C to A and G to T mutations. The resulting adjusted data were then screened for mutation peaks in excess of 7% as compared to the wild type peak, and each reported assay was individually analyzed and assessed for validity using criteria such as extension rate, shape of peak area and location of peak area over the expected peak position.

### *In vivo* Tumorigenesis Assay

FACS-sorted CD133+/EpCAM+ and CD133−/EpCAM+ cells isolated from P_1_ (namely, the 2^nd^ generation in mice) xenograft tumors were mixed with Matrigel (BD, 1∶1 volume ratio) and implanted subcutaneously in NOD/SCID mice (The Jackson Laboratory) to analyze their ability to initiate tumors. Tumor volumes, which were calculated as length×width×height×0.5, were measured on day 84 post-implantation and are shown as the mean±SEM (n = 5).

### *In vivo* Limiting Dilution Assay

The differential tumorigenic potentials of the cultured CSCs and their differentiated progeny were determined in SCID-bg mice (The Jackson Laboratory) by injecting titrated numbers of cells mixed with Matrigel at a 1∶1 ratio. Tumor volumes were measured at regular intervals for a period of 63 days and plotted as the mean ± SEM (n = 5).

### *In vivo* Antitumor Efficacy Evaluation

Tumor growth inhibition studies were performed in SCID-bg mice. Briefly, 2×10^6^ viable dissociated CSCs were subcutaneously inoculated, and mice bearing ∼200 mm^3^ tumors were randomly divided into three groups and were treated with 10 mg/kg PF-04691502 (PO, QD×14; n = 8) or vehicle (0.5% methylcellulose in water, PO, QD×14; n = 8). Tumor volumes are shown as the mean ± SEM. Relative change of body weight (RCBW; %) was calculated as the product of (BWi–BW0)/BW0×100%. BWi was the body weight on the day of dosing, and BW0 was the body weight on the first day of the treatment.

### Western Blot Analysis

Vehicle- or PF-04691502-treated tumors were flash frozen with liquid nitrogen. Frozen tumors were homogenized and lysed with 1× cell lysis buffer (Cell Signaling Technologies). Lysates were cleared of cell debris by centrifugation at 14,000 rpm for 30 minutes at 4°C, the supernatants were collected, and protein levels were quantified by the BCA protein assay (Pierce). Equal amounts of protein were resolved by polyacrylamide gels. After transferring the protein to nitrocellulose membranes, the membranes were blotted with the primary antibody pAKT (Ser473; Cell Signaling Technologies) or α-tubulin (Sigma). Secondary antibodies were purchased from Amersham Biosciences. Bands were detected by chemiluminescence with the SuperSignal West Dura Substrate (Pierce), and images were captured with an AlphaImager system.

### Statistical Analysis

All analyses were performed using GraphPad Prism (V.5.00 for Windows). Values of p<0.05 were considered statistically significant.

## Results

### Establishment of PDX Tumor Models and Confirmation of CD133+/EpCAM+ Tumorigenic CSC Population

**Figure 1:**
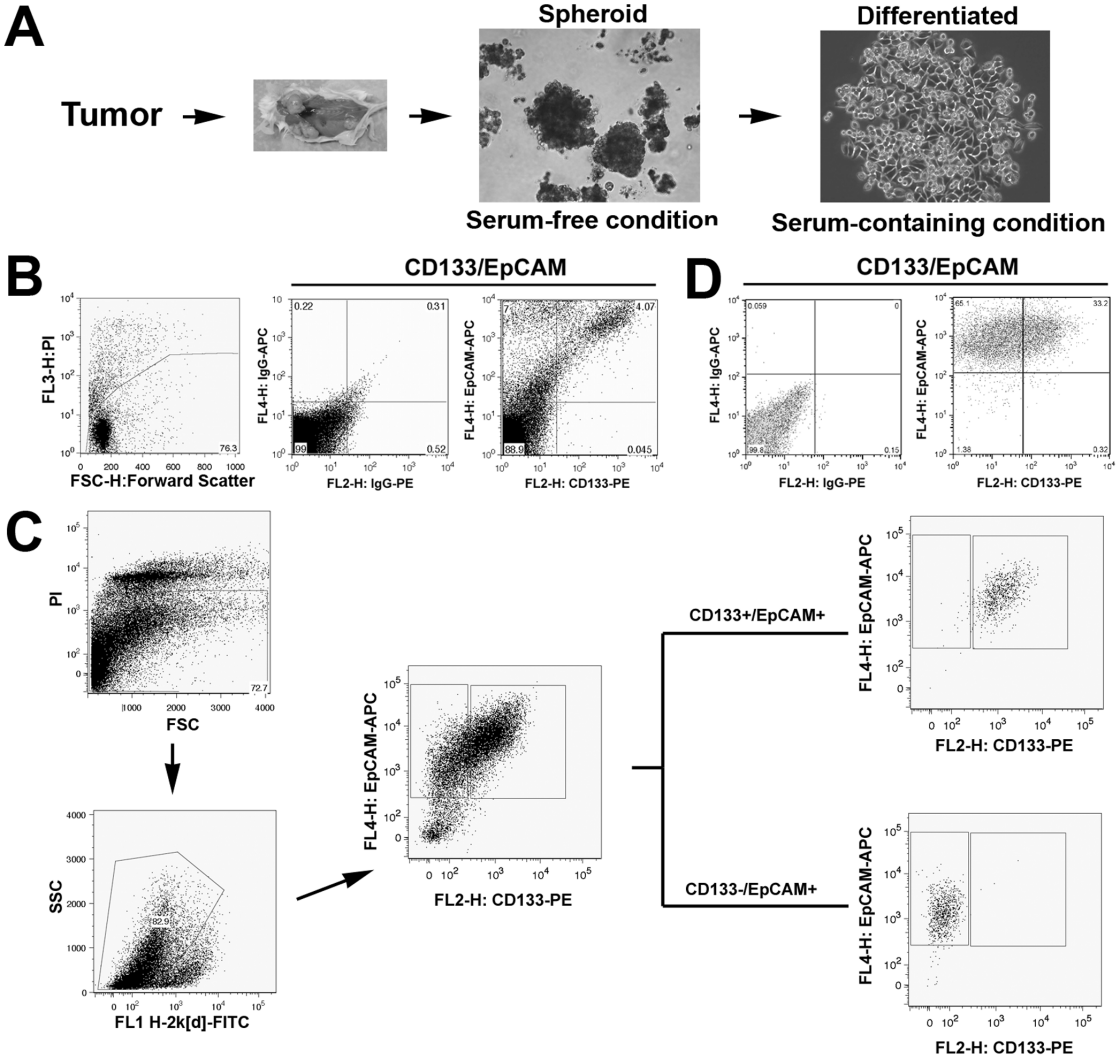
Culture of spheroid CSCs and identification of CD133+/EpCAM+ cells. *A*, Schema illustrating the work flow of spheroid CSC generation and differentiated cell populations as an experimental system, including the transplantation of a patient tumor into NOD/SCID mice, propagation of CSCs from P_1_ xenograft tumors (spheroid; 10× objective magnification), and differentiation of CSCs into adherent cells with epithelial morphology (differentiated; 20×). *B*. Flow cytometric analysis of the primary cells isolated from the original patient tumor. PI staining excludes the dead cells (a). APC- and PE-conjugated isotype controls are shown in (b). A population of CD133+/EpCAM+ cells was detected (c). *C*. FACS of CD133+/EpCAM+ colon CSCs from the primary cell population derived from P_1_ xenograft tumors. Dead cells and murine cells were first excluded by PI staining and using an anti-mouse specific monoclonal antibody H-2k[d], respectively (a & b). CD133+/EpCAM+ and CD133−/EpCAM+ populations were gated according to the baselines of isotype controls and sorted (c). Finally, enrichment of both populations in the sorted samples was confirmed by flow cytometry (d & e). *D*. Expression of the CSC markers in a fraction of cultured spheroid CSCs (right panel). Left panel shows isotype controls.

Fragments of freshly resected tumor sample from a colorectal cancer patient were subcutaneously implanted in two NOD/SCID mice to establish a colorectal cancer PDX model, which was then used to generate spheroid CSCs in culture (workflow for the generation of spheroid CSCs shown in Fig. 1A). Simultaneously, freshly dissociated primary cells from patient samples were assessed for the expression of colon CSC markers CD133/EpCAM by flow cytometry (Fig. 1B). After excluding non-viable cells by propidium iodide staining (Fig. 1B-a), approximately 4–6% CD133+/EpCAM+ cells were detected in the tumor tissue (Fig. 1B-c), suggesting the presence of putative CSCs.

**Figure 2:**
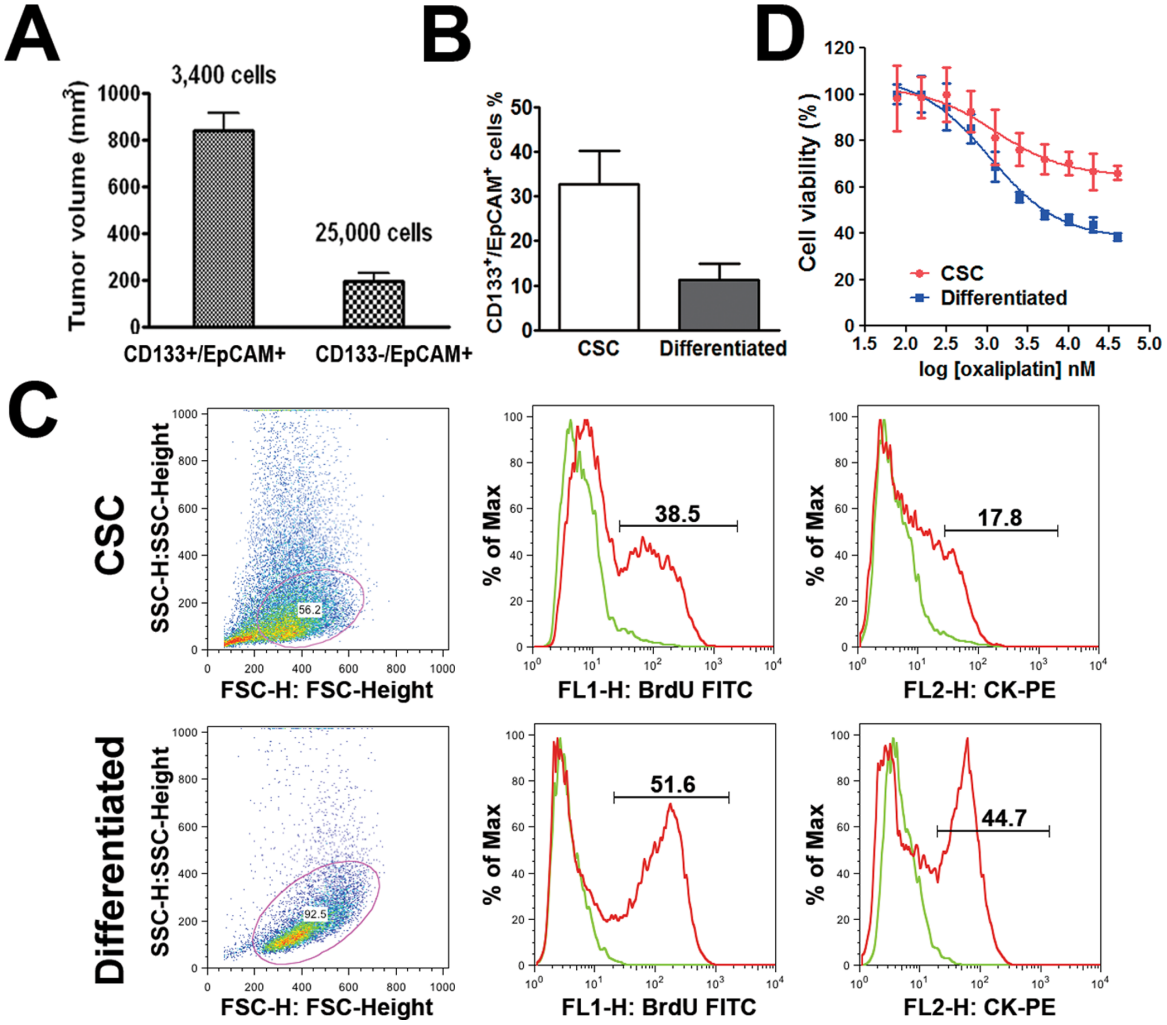
Enhanced tumorigenic potential of CD133+/EpCAM+ CSCs, CSC differentiation and drug resistance. *A*. Average tumor volumes in NOD/SCID mice inoculated with CD133+/EpCAM+ (3,400 cell/animal; putative CSCs) or CD133−/EpCAM+ (25,000 cell/animal; putative differentiated) cells after 12 weeks of implantation (mean±SEM; n = 5). *B*. Percentage of CD133+/EpCAM+ expressing cells in the CSC and differentiated cell populations (mean ± SEM, n = 3; unpaired, two-tailed student t-test, P<0.05). *C*. Representative results of cell proliferation rates and expression levels of cytokeratin in CSCs and differentiated cells. Cell numbers on Y axes are adjusted as a percentage of the maximum cell numbers analyzed. *D*. Representative data showing the *in vitro* drug sensitivity of CSCs and their differentiated progeny in response to oxaliplatin as assessed by CellTiter Glo® assay.

When P_1_ xenograft tumors reached 500–700 mm^3^, the tumor tissues were collected and processed for flow cytometric analysis and cell sorting. Flow cytometric analysis showed that the percentage of CD133+/EpCAM+ cells in P_1_ xenograft tumors remained in the same range as that in the primary tumor. Dissociated single P_1_ tumor cells were further sorted by FACS. After the exclusion of non-viable cells (Fig. 1C-a) and murine cells (Fig. 1C-b) as described in Materials and Methods, CD133+/EpCAM+ and CD133−/EpCAM+ cell populations were isolated (Fig. 1C-c). Sorted cell populations were enriched for CD133+/EpCAM+ (∼98%; Fig. 1C-d) or CD133−/EpCAM+ (∼96%; Fig. 1C-e) cells. Both cell populations were immediately mixed with Matrigel (at a 1∶1 volume ratio) and implanted subcutaneously in NOD/SCID mice to determine their tumorigenic potential. Based on the feasible cell numbers, CD133+/EpCAM+ and CD133−/EpCAM+ cells were implanted in five NOD/SCID mice at 2,400 and 25,000 cells per animal, respectively. Palpable tumors were first detected in CD133+/EpCAM+ group in 6 weeks and then detected in the CD133−/EpCAM+ group at 7 weeks post-implantation. When the animals were sacrificed at week 12, the volume of the tumors derived from CD133+/EpCAM+ cells was significant larger than that derived from CD133−/EpCAM+ cells (Fig. 2A; P<0.01). These results confirm that colorectal cancer consists of a small population of tumor-initiating CSCs, which are positive for CD133 and EpCAM expression.

### Generation of Stable Spheroid Cultures of CSCs

After culturing primary cells isolated from P_1_ xenograft tumors in serum-free stem cell medium, non-adherent 3-dimensional spheroids were observed within 2–3 weeks (Fig. 1A), and further expansion of the culture generated a large amount of spheroids within 2 months. Flow cytometric analyses of cultured spheroid cells show that CD133+/EpCAM+ cells composed 33.2% of the total cells (Fig. 1D; right panel), suggesting that CD133+/EpCAM+ cells were enriched approximately 5-fold in tumor spheroid culture compared to 4–6% of CD133+/EpCAM+ cells in both the original patient sample and the xenograft tumors (Fig. 1B and data not shown, respectively). Tumor spheroid cells were continually cultured *in vitro* over the past 2.5 years without showing a significant change in CD133/EpCAM expression levels or signs of senescence. The CSC properties of tumor spheroid cells were demonstrated in the experiments described below. Tumor spheroid cells are thereby referred to in the text as CSCs.

### Relatively Quiescent, Undifferentiated State of CSCs

To induce the differentiation of CSCs, tumor spheroid cells were dissociated into single cells and cultured in serum-containing media as described in the Materials and Methods. Adherent cells with epithelial morphology appeared within 1–2 days (Fig. 1A) and continued to propagate rapidly for 2–3 months until proliferation halted. Consistent with our earlier finding in the CSC population directly derived from a patient sample [23], a reduction of the CD133+/EpCAM+ cell fraction was observed in the adherent, differentiated cells (11.2%, Fig. 2B).

It has been implicated that CSCs retain a quiescent state. We assessed cell proliferation rates by using a BrdU flow cytometry kit. The results showed that the enriched CSC population contained ∼38.5% BrdU-positive cells compared to ∼51.6% in the differentiated cells. Reduced BrdU incorporation in the CSC population indicates that these cells were relatively quiescent compared to differentiated cells (Fig. 2C). Similarly, the fraction of cytokeratin-expressing cells in CSCs was smaller (∼17.8%) compared to that in the differentiated cell population (∼44.7%; Fig. 2C). As cytokeratin is a general marker for epithelial differentiation, lower levels of cytokeratin expression in CSCs are indicative of their relatively undifferentiated state.

### Drug Resistance of CSCs

*In vitro* cell viability assays revealed that, compared to differentiated cells, CSCs exhibited substantial resistance to oxaliplatin (Fig. 2D; difference in linear regression slope; P<0.01). Our finding is in agreement with previous reports showing the drug resistant nature of CSCs [24]–[26], suggesting that these cells might be responsible for clinical recurrence in cancer patients after chemotherapy.

### Tumorigenicity and Tumor Development of CSCs *in vivo*

**Figure 3:**
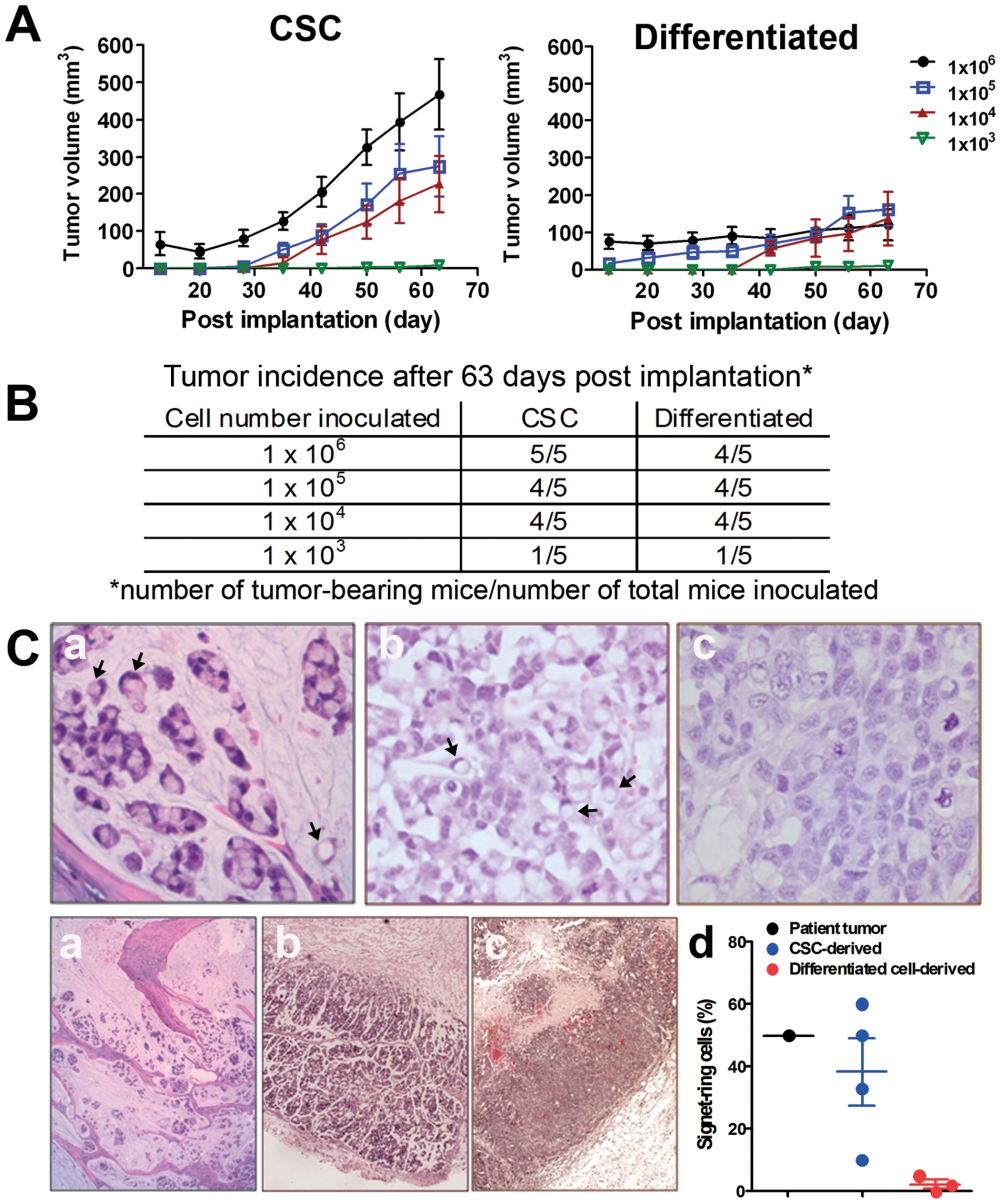
Enhanced tumorigenic potential of CSCs and histology of xenograft tumor tissues. *A*. Tumor volumes in the *in vivo* limiting dilution assay for CSCs and their differentiated progeny in SCID-bg mice as described in the Materials and Methods (mean ± SEM; n = 5). Differences in the growth rates were found between the CSC and differentiated cell groups inoculated with higher cell numbers (1×10^6^ and 1×10^5^; slope difference in lineage regression; P<0.01). *B*. Tumor take rates after 63 days post-implantation for CSCs and differentiated progeny. *C*. H&E stained sections of the primary patient tumor (*a*) and xenograft tumors derived from CSCs (*b*) and differentiated cells (*c*; upper panel, 40×; lower panel, 4×). Signet-ring cells were frequently identified in both patient and CSC-driven tumors and are indicated by arrows (a and b at 40×). A dot plot shows the frequency of signet-ring cells identified in the patient tumor, the CSC-derived xenograft and the differentiated cells-derived xenograft (d).

Varying amounts (i.e., 10^3^, 10^4^, 10^5^ and 10^6^ cells/animal) of dissociated CSCs and differentiated cells were inoculated subcutaneously in five SCID-bg mice. The tumor take rates between the two groups appeared to be similar (Fig. 3B); however, the mean tumor volumes were dramatically larger in the mice implanted with 1×10^5^ or 1×10^6^ CSCs than in the groups injected with the same number of differentiated cells (Fig. 3A; difference in linear regression slope, P<0.01). These results suggest that CSCs are more tumorigenic compared to their differentiated cell progeny.

### Recapitulation of Patient Histopathology and Frequency of CD133+/EpCAM+ Cells in Xenograft Tumors

Xenograft tumors derived from CSCs and differentiated cells were processed for histological examination to compare to the morphology of the primary patient tumor. Signet-ring cells were frequently identified in both the patient tumor (Fig. 3C-a) and the CSC-derived xenografts (Fig. 3C-b). Signet-ring cells contain a single cytoplasmic vacuole, which eccentrically displaces the nucleus (marked by arrows). In contrast, signet-ring cells were almost absent in the differentiated cell-derived xenografts (Fig. 3C-c). Based on the assessment of a board-certified pathologist, the percentages of the signet-ring cells were approximately 45%, 38% and 1% for the patient tumor, CSC-derived xenograft and the differentiated cell-derived xenograft, respectively (Figure 3C-d). These results indicate that xenograft tumors originating from the CSC population better recapitulate the histological features of the original patient tumor.

**Figure 4:**
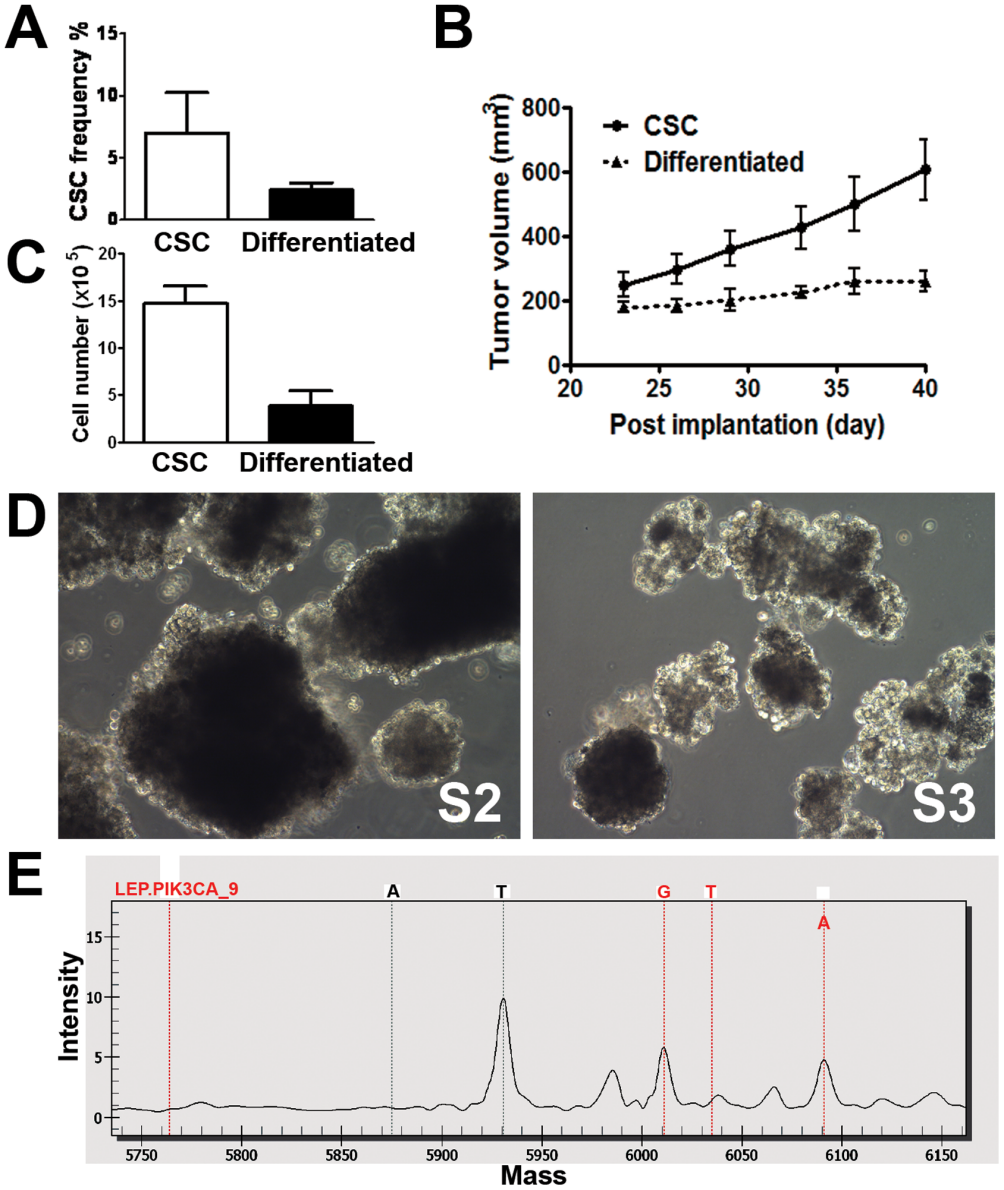
Self-renewal of CSCs and mutation detection. *A*. Flow cytometric analysis of the CD133+/EpCAM+ fraction in xenograft tumors generated by CSCs and differentiated progeny. Data are shown as the CSC frequency representing the percentage of CD133+/EpCAM+ cells in tumors of two distinct origins (mean ± SEM, n = 3; unpaired, two-tailed student t-test, P<0.05). *B*. Re-implantation of tumor fragments obtained from CSCs or differentiated cell-derived xenograft tumors in secondary SCID-bg mice. Tumor volumes are shown as the mean ± SEM (n = 5). The difference in the growth curves between the two groups is statistically significant (difference in slope by linear regression; P<0.05). *C*. Cell proliferation of the primary cells isolated from either CSCs or differentiated cell-derived xenograft tumors in the stem-cell culture condition. Viable cells were plated at 20,000 cells per well and cultured for 17 days. Cell numbers were manually counted, and total cell counts are shown as the mean ± SEM (n = 6; unpaired, two-tailed student t-test, P<0.01). *D*. Spheroid CSC cultures re-established under the stem cell conditions from xenograft tumors generated by the serial implantation of CSC-derived xenograft tumors. P_2_, CSCs cultured from xenograft tumors derived from the original CSCs. P_3_, CSCs cultured from xenograft tumors derived from the P_2_ CSCs. *E*. Partial MALDI-TOF mass spectrum showing the H1047R mutation in *PIK3CA*. The red dotted lines indicate, from left to right, the unextended primer peak, the 3 potential peaks for the mutant G or T alleles and the wild type A allele. The high peak in the middle of the figure is a T allele of *PDGFRA* in the same MALDI-TOF mass spectrum.

Before implantation in mice, the percentages of cells expressing CD133+/EpCAM+ markers were higher in the CSC-enriched population (33.2%) than in differentiated cells (11.2%; Fig. 2B). Despite the higher percentages of CD133+/EpCAM+ cells in the cultured CSC population, the percentage of CD133+/EpCAM+ cells in the xenograft tumors declined to 4–6%, which is consistent with the percentage of CD133+/EpCAM+ cells observed in the original patient tumor (Fig. 1Bc and Fig. 4A). In contrast, ∼2% CD133+/EpCAM+ cells were observed in the differentiated cell xenograft tumor (Fig. 4A). These results suggest that cultured CSCs differentiate upon implantation.

### Self-renewal Capacity of CSCs

The ability to self-renew, a key property of CSCs, was assessed by the serial transplantation of xenograft tumors, as described in the Materials and Methods. Briefly, xenograft tumors derived from CSCs or differentiated cells were removed from tumor-bearing mice. Tumor fragments (2–4 mm^3^) were implanted subcutaneously into secondary SCID-bg mice. Xenograft tumors (designated as P_2_ xenograft tumors) originating from differentiated cell tumor fragments grew slowly compared to tumors derived from CSC tumor fragments (Fig. 4B). The results of the above serial implantation experiment suggest that CSC origin tumors grow more aggressively in conjunction with a larger percentage of CSCs within the cell/fragment implant. Accordingly, the *in vitro* cultivation of primary tumor cells derived from the xenograft tumor originating from CSCs exhibited a higher proliferative capability under stem cell conditions compared to those isolated from the tumors originating from differentiated cells (Fig. 4C). These results indicate that primary cells from CSC-derived tumors may have a growth advantage *in vitro* most likely due to their higher content of CSCs.

Spheroid CSCs (designated as S_2_) were sequentially established from P_2_ xenograft tumors (Fig. 4D). When implanted in mice, S_2_ CSCs consistently formed rapidly growing P_3_ xenograft tumors (data not shown). S_3_ CSCs were also re-established from primary cells generated from P_3_ tumors (Fig. 4D). Both S_2_ and S_3_ CSCs contained CD133+/EpCAM+ cells at levels comparable to the parental CSCs (30–40%; data not shown). The above results suggest that *in vitro* cultured colorectal CSCs are capable of self-renewal.

### Analysis of Common Oncogenic Mutations in CSCs and Biomarker Discovery

To identify predictive biomarkers that can potentially guide targeted therapy, we conducted genetic analysis with the OncoCarta™ panel using DNA isolated from cultured CSCs and differentiated cells. Mutational analysis of 19 common oncogenes, including *ABL1, AKT1, AKT2, BRAF, CDK, EGFR, ERBB2, FGFR1, FGFR3, FLT3, JAK2, KIT, MET, HRAS, KRAS, NRAS, PDGFRα, PIK3CA,* and *RET*, was conducted. Interestingly, only the *PIK3CA (H1047R)* mutation was identified in both cell types (Fig. 4E).

### Profound Antiproliferative Activity of PF-04691502 in Mutant CSCs

**Figure 5:**
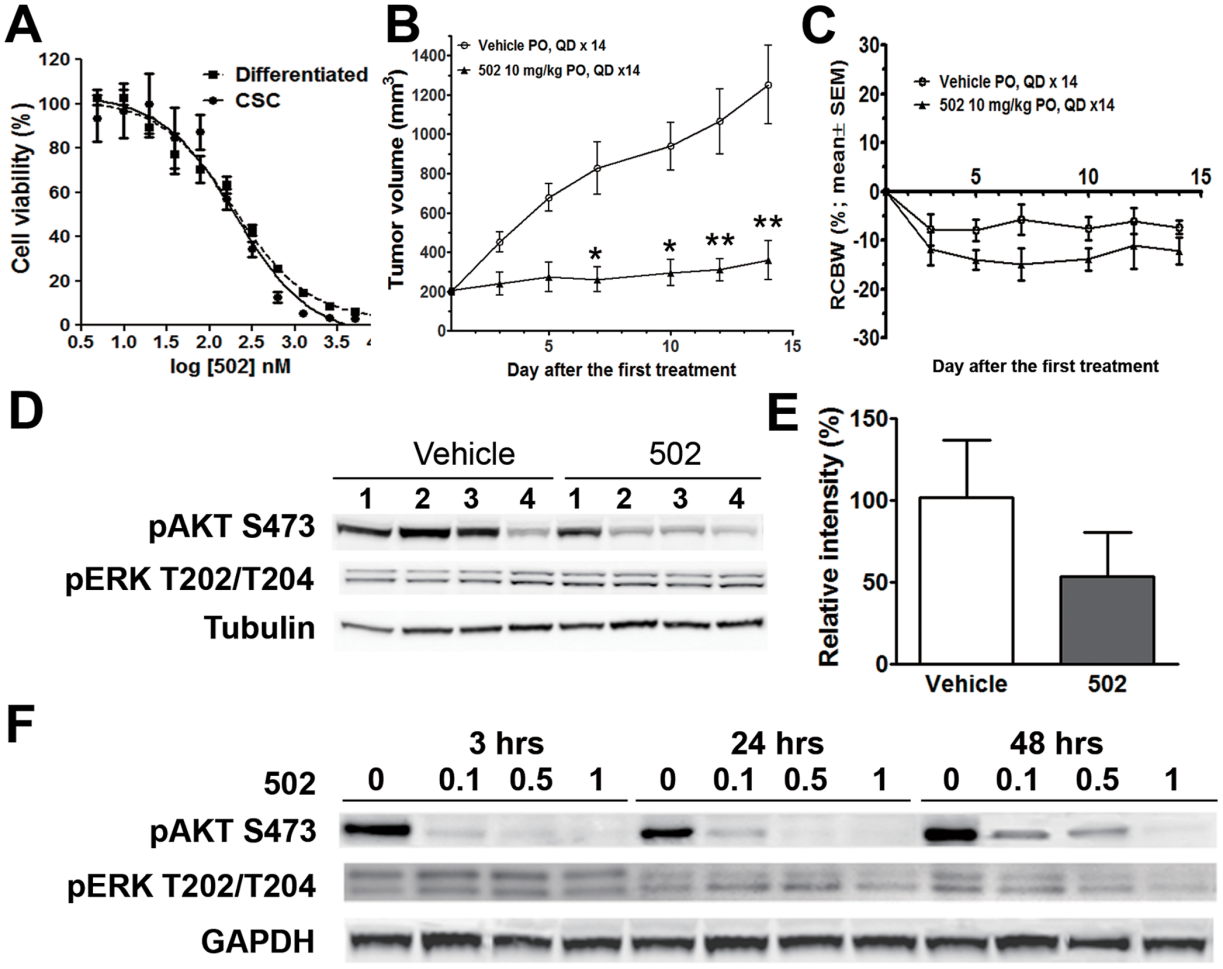
Antiproliferative and antitumor activities of PF-04691502 through the modulation of the PI3K/mTOR pathway. *A*. Representative dose-dependent responses of CSCs and differentiated cells after treatment with PF-04691502. Cell viability, as measured by CellTiter Glo® assay, is shown as the mean ± SEM (n = 5). *B*. Tumor growth inhibition induced by the treatment with PF-04691502 in SCID-bg mice in comparison with the vehicle controls. *P≤0.05; **P≤0.001 (two-way ANOVA analysis). *C.* Relative change in body weight of mice treated in the experiment (%). *D.* Western blot analysis of treated xenograft tumors. The expression levels of pAKT (S473), pERK (T202/T204), and the loading control α-tubulin are shown in four individual xenograft tumors in each treatment group. *E.* Quantitative data of the expression levels of pAKT (S473) relative to the loading controls (mean ± SEM; n = 4). F. CSCs were treated with PF-04691502 (502) at 0.1, 0.5 and 1 µM in vitro for 3, 24, and 48 hours. The changes in pAKT (S473) and pERK (T202/T204) were evaluated by Western Blot. GAPDH served as a loading control.

We then determined the *in vitro* antiproliferative activity of PF-04691502, a potent dual inhibitor of all PI3K isoforms and mTOR (TORC1 and TORC2). Indeed, PF-04691502 exhibited a significant inhibitory effect on the cell proliferation of CSCs (Fig. 5A). Interestingly, unlike oxaliplatin (see Fig. 2D), PF-04691502 showed a similar inhibitory effect on both mutant CSCs and their differentiated progeny (IC_50_ = 202 nM and 195 nM, respectively), suggesting that the antiproliferative activity of PF-04691502 may be associated with the *PIK3CA (H1047R)* mutation identified in both cell populations.

Western blot analysis was performed to assess the biological effect of PF-04691502 *in vitro*. The spheroid CSCs were treated with PF-04691502 at 0.1, 0.5 and 1 µM for a desired treatment duration. As shown in Fig. 5F, PF-04691502 at all tested concentrations induced a robust inhibition of pAKT (S473). After a longer treatment (48 hrs), the pAKT level showed a slight recovery in the 100 nM- or 500 nM-treated cells but remained at the levels lower than the control level. pERK (T202/T204) remained unchanged in all tested conditions.

### Tumor Growth Inhibition of PF-04691502 in Xenograft Models Derived from CSCs Harboring the *PIK3CA (H1047R)* Mutation

Mice bearing xenograft tumors (∼200 mm^3^) were subjected to treatment with PF-04691502 or vehicle. Daily oral administration of PF-04691502 (10 mg/kg, QD) was tolerated throughout the experiment (Fig. 5C). None of the PF-04961502-treated mice exhibited body weight loss of over 20% until the end of the study, at which time the average tumor volume in the vehicle control group reached 1,000 mm^3^. Compared to the vehicle treatment, PF-04961502 markedly inhibited xenograft growth (Fig. 5B). Significant tumor growth inhibition was first observed on Day 7 of the treatment and was sustained until Day 14 (Fig. 5B; P<0.05). The average tumor volume was significantly smaller in the PF-04691502 treatment group (360±35 mm^3^) than in the vehicle-treated group (1252±71 mm^3^; P<0.01) at the study end point (Day 14).

### Reduction of pAKT (S473) Levels in Xenograft Tumors Treated with PF-04691502

Pharmacodynamic analysis was performed by Western blotting using tumor tissues collected during the above efficacy studies. The results showed a significant inhibition of pAKT (S473) in PF-04691502-treated mice compared to vehicle-treated mice (Fig. 5D-E). The change of p-ERK (T202/T204) level was not observed.

## Discussion

One of the reasons for the high attrition rate in cancer drug discovery is the lack of preclinical animal models that can recapitulate the disease biology in patients. Derived directly from clinic tumor samples, PDX tumor models faithfully maintain the heterogeneity of clinical patient tumors and provide a clinically relevant tumor model for drug testing [27]. As such, PDX models provide a convenient platform from which to study CSC biology and to develop CSC-relevant tumor models [27].

The discovery of tumor-initiating CSCs has important therapeutic implications. The CSC hypothesis states that these tumor-initiating cells go through a long period of dormancy and often survive despite standard chemotherapy, potentially leading to disease relapse. It is crucial to develop drug candidates that target CSCs in order to enhance current cancer therapy. As cancerous malignancies contain a heterogonous population of tumor cells, a therapeutic agent should not only target an oncogenic pathway driven by somatic mutations but also inhibit the proliferation of the tumor-initiating CSCs harboring the mutations. Herein, we report the development and validation of a CSC population from a colorectal PDX model and its application in the evaluation of an experimental drug targeting the mutation harbored by the CSCs.

In addition to CD133 and EpCAM, distinct cell surface markers (including CD44 and CD26) help delineate tumor-initiating CSCs in colorectal cancer tissues [28], [29]. Identifying CSCs with specific cell surface markers is critical for understanding the involvement of CSCs in disease progression and the subsequent development of therapeutic options. To this end, and in agreement with earlier reports [13], [14], we first demonstrated that CD133+/EpCAM+ tumor cells are tumor-initiating CSCs in the studied patient colorectal tumor by conducting the tumorigenesis assay in immunodeficient mice (Fig. 2A). Simultaneously, patient tumor fragments were implanted in immunodeficient mice to establish a PDX tumor model.

Despite the caveats that the experimental approaches involve artificial conditions, in vitro cultures of CSCs were a faster and more economical approach to obtaining a large quantity of CSCs for drug testing purposes [30]. In this report, we used the established PDX model at an early passage to initiate CSC propagation, resulting in a tumor spheroid culture enriched with CD133+/EpCAM+ cells. In the primary patient tumor, the CD133+/EpCAM+ cell fraction only composed 4–6% of the total cell population (Fig. 1Bc). However, the cultured tumor spheroid cells consisted of 33.2% of CD133+/EpCAM+ cells, which is approximately 5-fold higher than the portion in the original patient tumor tissue (Fig. 1D, right panel). In agreement with previous reports, our results show that colorectal CSCs can indeed be maintained and further enriched under stem cell culture conditions [14], [23], [31].

Exposure to serum-containing media induced the morphological differentiation of tumor spheroid cells into adherent monolayer cells (Fig. 1A), accompanied with the reduced percentage of CD133+/EpCAM+ cells (from 33.25 to 11.2%; Fig. 2B). Because of their high content of CD133+/EpCAM+ cells, tumor spheroid cells were proved to be highly tumorigenic upon xenotransplantation compared to their differentiated progeny when equivalent amount of cells were injected (Fig. 3A). Thus, the tumor spheroid cells fulfilled the important CSC criterion of being capable of tumorigenesis upon transplantation, of CSCs defined by earlier publications [16].

The resistance of CSCs to conventional cytotoxic agents has been well documented in various tumor types in both preclinical [24], [25] and clinical [26], [32] settings. In our study, we obtained similar results to those of previous studies by treating spheroid CSCs and differentiated cells with oxaliplatin (Fig. 2D). Importantly, tumor fragments derived from CD133+/EpCAM+ tumor spheroid cells, but not from CD133−/EpCAM+ differentiated cells, demonstrated continual growth potential after re-implantation in the secondary mice (Fig. 4B). Similarly, primary tumor cells originating from the CD133+/EpCAM+ spheroid CSC-derived xenograft tumors exhibited a higher proliferative potential under the stem cell conditions compared to those from CD133−/EpCAM+ cell-derived xenograft tumors (Fig. 4C). These proliferating cells went on to form tumor spheroids enriched with CD133+/EpCAM+ cells (Fig. 4D). These results suggest that tumor spheroid CSCs are capable of greater self-renewal and propagation both *in vitro* and *in vivo*.

The histopathology of xenograft tumors derived from CSCs, but not differentiated cells, more closely resembled the characteristics of the original patient tumor by displaying signet-ring cell features (Fig. 3C). Differentiated cells also gave rise to a tumor, but the resulting tumor failed to faithfully recapitulate the histopathological features of the original patient tumor. Earlier studies have demonstrated that CSCs are able to form xenograft tumors with histology resembling the original patient tumors [33], [34]. One of the reports showed that, in ovarian cancer, both CD133+ (CSCs) and CD133- (non-CSCs) cells gave rise to histologically identical xenograft tumors [33]. Further studies are required to elucidate whether CSCs give rise to xenograft tumors more closely resembling the clinical tumor than non-CSCs. Our results further highlight the importance of developing CSC-relevant tumor models representing actual clinical lesions for the pharmacological characterization of experimental drugs in preclinical studies. Overall, we demonstrate that CD133+/EpCAM+ tumor spheroid cells meet the key criteria of CSCs and may serve as a CSC model for both the *in vitro* and *in vivo* testing of experimental drugs.

Intriguingly, the spheroid CSC population consisting of approximately 30% CD133+/EpCAM+ cells gave rise to xenograft tumors that contained only 4–6% of CD133+/EpCAM+ cells (Fig. 4A). The latter percentage is consistent with that observed in the original patient tumor (Fig. 1Bc). A possible explanation for this finding is that tumor infiltrating stromal cells diluted the percentage of CD133+/EpCAM+ cells. However, as murine cells have been excluded by using a species-specific antibody during flow cytometric analysis, CD133+/EpCAM+ cells most likely differentiate into CD133- and/or EpCAM-negative cells upon xenotransplantation. Thus, a lower fraction of CD133+/EpCAM+ CSCs was observed in xenograft tumor tissues. By using single cell-derived cultures, previous studies have demonstrated that colorectal CSCs possess multilineage differentiation capability [31]. Presumably, in our studies, the loss of CD133+/EpCAM+ cells during both *in vitro* differentiation induction and *in vivo* xenotransplantation is due to the differentiation of CSCs. Importantly, a consistent percentage of CSCs in a particular patient tumor and the derived xenograft tumors seems to be precisely regulated through controlled differentiation mechanisms.

Further identification of a *PIK3CA (H1047R)* (but not *KRAS*) mutation in both spheroid CSCs and the differentiated progeny by using the OncoCarta™ panel implicates a therapeutic opportunity for PF-04691502, a potent inhibitor of the PI3K/mTOR pathway. In colorectal cancer, inappropriate activation of PI3K signaling occurs frequently. The *PIK3CA* gene codes for the catalytic subunit p110α of PI3Ks and is frequently mutated in various human cancers including colorectal cancer with a frequency of ∼30% [35]–[37]. The most frequently affected sites are E542 and E545 in the helical domain and H1047 in the catalytic domain. Although all of these mutant forms of p110α have been demonstrated to increase oncogenic potential, the H1047R mutation is associated with higher cell proliferation and invasion, greater survival capability during growth factor deprivation stress, chemo-resistance and resistance to EGFR-targeted therapies [38]–[45]. Colorectal cancer patients harboring the activating somatic *PIK3CA* mutation have the worst clinical outcomes [42]. Furthermore, evidence that the PI3K pathway regulates the differentiation of spheroid colorectal CSCs has been previously reported [31]. More interestingly, the crucial role of the PI3K pathway in colorectal CSCs was discovered through the induced differentiation of spheroid CSCs into adherent cells by using a small molecule library [31]. Therefore, therapeutics targeting the PI3K pathway can potentially benefit the subpopulation of colorectal cancer patients harboring the *PIK3CA* mutation.

Earlier studies have shown that *PIK3CA* mutations are a predictive marker for responses to PI3K and mTOR inhibitors [3]–[6]. Conversely, coexisting *KRAS* mutations confer the resistance of PIK3CA mutant colorectal cancer to PI3K/mTOR inhibitors in preclinical [3] and in clinic studies [4]. In our study, no *KRAS* mutation was identified in the cultured CSCs by the OncoCarta™ panel, leading to CSC sensitivity to PF-04691502 treatment. Our data confirm that the sensitivity of *PIK3CA* mutant colorectal cancer to PI3K/mTOR inhibitors may depend on the presence of wild-type *KRAS* gene [4]. Alternatively, although CSCs appeared to be resistant to the cytotoxic agent oxaliplatin (Fig. 2D), PF-04691502 significantly inhibited the *in vitro* proliferation of colorectal CSCs (Fig. 5A) and tumor growth in xenograft tumor models derived from CSCs in mice (Fig. 5B). The data are in agreement with an earlier report showing that AKT activation is important for the *in vivo* tumor growth of colorectal CSCs [46]. Targeting the PI3K/mTOR pathway may achieve anti-CSC activity. Our results not only confirm that *PIK3CA* mutant tumors are sensitive to treatment with PI3K/mTOR inhibitors but also further elucidate the ability of PI3K/mTOR inhibitors to target mutant CSCs in colorectal cancer.

In contrast to cytotoxic agents, PF-04691502 equally inhibited the *in vitro* proliferation of both CSCs and their differentiated progeny. PF-04691502 targets PI3K/mTOR genes. Presumably, the equivalent effectiveness on colorectal CSCs and differentiated cells is related to the presence of the *PIK3CA* mutation in both cell types, and thus, both CSCs and differentiated cells are similarly affected by the inhibitor. Inhibiting the proliferation of both cellular populations is a preferable anticancer approach because neighboring differentiated tumor cells might contribute to the formation of the CSC niche in which CSCs reside [30]. Theoretically, disrupting the CSC niche could lead to impaired tumor growth (curative treatment is uncommon in colon cancer). Normal colorectal stem cells carrying the wild-type *PIK3CA* gene will not be affected by the inhibition of the PI3K/mTOR pathway by PF-04691502, which represents a therapeutic window for PF-04691502 treatment in colorectal cancer. However, this hypothesis needs to be tested once the normal stem cells become available for study.

In line with an earlier study using another PI3K inhibitor [3], the inhibition of AKT (S473) phosphorylation by PF-04691502 has been well established as a biomarker for targeted inhibition of the PI3K/mTOR pathway [11]. In this study, we also observed the inhibition of pAKT (S473), suggesting that the *in vitro* growth inhibition and the *in vivo* efficacy of PF-04691502 was mediated by impairing the PI3K/mTOR pathway. The work reported by Serra et al. [47] showed that a longer period (48 hours) of exposure to low doses of NVP-BEZ235, a dual PI3K/mTOR inhibitor, induced an increase in pAKT due to the negative feedback loop by disrupting mTORC1/S6K1 pathway. In our study, treatment of the spheroid CSCs with PF-04691502 at 1 µM sustained the pAKT suppression for up to 48 hrs. At the lower dose levels (i.e., 0.1 and 0.5 µM), substantial inhibition of AKT phosphorylation was observed for 24 hrs. The pAKT level recovered slightly after a longer treatment (48 hrs), but still remained below the control level. This data implicates for a moderate negative feedback effect by impairing PI3K/mTOR signaling in the spheroid CSCs. pERK levels remained unchanged regardless the doses and treatment durations. In comparison, the work by Yuan and colleagues demonstrated that PF-04691502 at high or low doses induced strong feedback effect in other tested cell lines, i.e, the pAKT level rebounded and exceeded the control level [11]. An activation of pERK was seen in some tested cell lines after PF-04691502 treatment, indicating a cell line dependent on activation of MAPK pathway [11]. Taken together, PI3K/mTOR inhibitors relieve differential feedback activation of AKT and the degree of the feedback highly depends on the cell type or the individual compound property. Therefore, for the clinical development of PF-04691502 or other PI3K/mTOR inhibitors, it is important to stratify the patient populations and also to have a rational combination strategy to overcome the disadvantages (i.e., negative feedback) of PI3K/mTOR inhibitor monotherapy.

In conclusion, we developed a CSC model from a colorectal cancer patient-derived xenograft tumor and identified a *PIK3CA (H1047R)* mutation in the CSC population as a predictive biomarker to guide targeted therapy. The therapeutic efficacy of a clinical candidate drug PF-04691502, a potent dual PI3K/mTOR inhibitor, was further demonstrated in both cell-based assays and in the xenograft tumor model derived from CSCs. The current study demonstrates the feasibility of developing novel tumor models with CSC properties to reveal the anticancer activity of a novel dual PI3K/mTOR inhibitor in colorectal cancers harboring a specific hotspot mutation, highlighting the importance of performing pharmacological studies using a genetically characterized CSC model. The results may assist in translating preclinical compounds into clinical trials by selecting an appropriate patient subpopulation.
